# Flavonoids as natural phenolic compounds and their role in therapeutics: an overview

**DOI:** 10.1186/s43094-020-00161-8

**Published:** 2021-01-20

**Authors:** Rakesh E. Mutha, Anilkumar U. Tatiya, Sanjay J. Surana

**Affiliations:** Department of Pharmacognosy, R. C. Patel Institute of Pharmaceutical Education and Research, Karwand Naka, Shirpur, Dist., Dhule, Maharashtra 425405 India

**Keywords:** Phenolics, Flavonoids, Secondary metabolites, Therapeutic action

## Abstract

**Background:**

Natural plants and plant-derived formulations have been used by mankind from the ancient period of time. For the past few years, many investigations elaborated the therapeutic potential of various secondary chemicals present in the plants. Literature revealed that the various secondary metabolites, viz. phenolics and flavonoids, are responsible for a variety of therapeutic action in humans.

**Main body:**

In the present review, an attempt has been made to compile the exploration of natural phenolic compounds with major emphasis on flavonoids and their therapeutic potential too. Interestingly, long-term intake of many dietary foods (rich in phenolics) proved to be protective against the development and management of diabetes, cancer, osteoporosis, cardiovascular diseases and neurodegenerative diseases, etc.

**Conclusion:**

This review presents an overview of flavonoid compounds to use them as a potential therapeutic alternative in various diseases and disorders. In addition, the present understanding of phenolics and flavonoids will serve as the basis for the next scientific studies.

## Background

Polyphenols is one of the major classes of naturally occurring compounds having at least one phenol group in their structure, present in the plants, including vegetables, fruits, cereals and dry legumes [1–3]. As these compounds show multiple physiological effects, when consumed as a component or dietary supplement, polyphenolics become a subject of interest in a scientific fraternity [4]. Polyphenols are secondary metabolites consisting of polyhydroxy phytochemicals of the plant kingdom and effective defense against pathogenic aggression and ultraviolet radiation [5]. These secondary compounds are biosynthesized through shikimic acid and phenylpropanoid pathways and believed to be participative in adapting the plants in a stressed situation due to environmental changes [6]. From this extensive class of polyphenolic compounds, more than 8000 have been already isolated, identified and described in detail [7].

In food, initially, polyphenols are used to manipulate astringency, bitterness, flavor, color, odor and oxidative stability. Throughout evolution in various plant lines, the ability to synthesize phenolic compounds was selected when these compounds met unique needs, allowing plants to cope with continuously evolving environmental conditions over evolutionary time [8]. Afterward, various epidemiological research activities and accompanying meta-analyses intensely recommended protection offered against the development of diabetes, cancer, osteoporosis, cardiovascular diseases and neurodegenerative diseases by long-term intake of plant polyphenols in our daily diet [9, 10]. These compounds, based on their chemical structures, are divided into various subclasses like phenolic acids, flavonoids, tannins, coumarins, lignans, quinones, stilbenes and curcuminoids [1].

Flavonoids are ubiquitously occurring polyphenolic compounds and comprise the broad class of natural products. To date, it has documented over 8000 different flavonoids and most of them are present in the cells or surfaces of various plant tissue organs [11]. A large variety of edible plant species contains flavonoids, which are considered to be important human dietary constituents [12, 13]. Flavonoids are among the most abundant and widespread secondary metabolites groups, which is extremely valuable to mankind, not merely because of their contribution to imparting plant colors, but also due to its several physiologically active members too [14]. The confirmation of flavonoid’s reliable positive benefits like cancer prevention has produced a considerable interest in research, including serving foods containing flavonoids [15].

## Main body

### Polyphenols and their therapeutic role

Epidemiological studies revealed an inverse relationship between the intake of polyphenolic rich food and threat of chronic human ailments [2, 9]. The generation of phenoxyl radicals via an acceptance of an electron by the phenolic groups of polyphenols creates the favorable disturbance in oxidative chain reactions inside cells [16]. Study revealed that polyphenols present in food and beverages exhibited an increase plasma antioxidant activity owing to accumulation of these reducing polyphenols, along with endogenous antioxidants in plasma, which, in turn, assist in absorption of iron like a pro-oxidative food component [2].

Consumption of polyphenol-rich diet protects lymphocytic deoxyribonucleic acid (DNA) from oxidative damage and work as antioxidants. A similar protective effect was evident for the beverages rich in polyphenols [17]. Polyphenols not only protect the cell and cellular components from oxidative damage but also reduce the risk of oxidative stress linked to different degenerative diseases [18–20].

#### Antidiabetic effect

Diabetes mellitus is a condition of physiological imbalance due to the alteration of various physiological parameters due to the impairment of the metabolism of glucose which resulted in hyperglycemia. Type 1 and type 2 are the two main categories of diabetes [21]. As diabetes is chronic, it leads to retinopathy, nephropathy and neuropathy, which may further result in blindness, kidney failure and amputations, respectively. Neuropathy may further create complications associated with sexual dysfunctions.

Scientific studies have revealed that polyphenols play an important role as an antidiabetic agent. Various catechin compounds from tea show antidiabetic action [22]. The potential of these secondary compounds as antidiabetic agents may be due to its inhibitory action in the gut for glucose absorption or by its peripheral tissue uptake. Diacetylated anthocyanins showed the antidiabetic effect at a dose of 1 mg/kg given with maltose as a source of glucose. This effect was not observed when it is given with glucose or sucrose [23]. Inhibition of α-glucosidase found in gut mucosa is responsible for such type of effect. Along with this, polyphenols have been investigated for their glucose transporter and intestinal glycosidase inhibitory activity [24].

A variety of polyphenols like isoflavones from soybeans, (−) epicatechin, epicatechin gallate, (+) catechin, (−) epigallocatechin, tannic acid, saponins, chlorogenic acid and compounds like glycyrrhizin from the liquorice root reduces S-Glut-1 mediated transport of glucose from the intestine. Owing to saponins, the transfer of the glucose from the stomach to the small intestine is delayed [25]. Stilbene compounds like resveratrol improvise whole-body glucose homeostasis and sensitivity to insulin in diabetic rats [26]. It also improves the status of diabetic nephropathy, dysfunction in the kidney and oxidative stress of diabetic rats. As resveratrol inhibits K^+^ adenosine triphosphate (ATP) and K^+^ (V) channel in beta cells considered to be a possible mechanism due to which effects like delaying of the onset of insulin resistance and decrease in insulin secretion [27]. Polyphenolic compound quercetin protects the lipid peroxidation and oxidative stress, which, in turn, helps in antidiabetic activity [28]. The antidiabetic potential of quercetin has been associated with its glucose uptake inhibitory phenomenon and modulation of mitogen-activated protein kinase pathway [29, 30]. One more study performed on *Hibiscus sabdariffa* extract proved that polyphenols (flavonoids, polyphenolic acids, protocatechuic acid and anthocyanins) lessen oxidative markers in the kidney, serum lipid profile and diabetic neuropathy [31]. Maize bran and vegetable contains ferulic acid, one of the polyphenolic compounds that helps in lowering lood glucose with an increase in insulin in plasma responsible for its strong antidiabetic activity [32]. Myricetin also showed strong glycemic control via insulin resistance amelioration and human pancreatic α-amylase inhibition [33]. In vivo study performed on diabetic rats elicits antidiabetic potential of resveratrol via intracellular glucose uptake stimulation and modulation of sirtuin-1 activity [34, 35]. The antidiabetic action of hispolon has been attributed with α-glucosidase and aldose reductase inhibitory action [36]. Gallic acid and p-coumaric acid also exhibited antidiabetic action via reduction in serum glucose level and rise in insulin level in diabetic rats [37]. The antidiabetic activity of cinnamic acid and caffeic acid was studied using mice and found effective through increase in glucose uptake and insulin sensitivity which results in reduction in glucose level [38, 39].

#### Anticancer effect

Various in vitro and in vivo studies of polyphenols were performed using human cancer cell lines. These studies concluded that polyphenols are protective and responsible for lowering tumor growth [40]. This type of beneficial effect was observed for various cancer sites, including the mammary glands, skin, lung and liver, and some sites of the digestive tract like the intestine, stomach and mouth. Despite the different mechanisms of action of different polyphenols, they show protective anticancer potential in few anticancer study models. Some of them are flavanones, isoflavones, catechins, ellagic acid, resveratrol, curcumin and red wine polyphenols [41].

Polyphenolic compounds show chemoprevention by several identified mechanisms like oxidation prevention, antiproliferation, detoxification of enzymes, initiation of apoptosis or cell cycle arrest, host immune system regulation, estrogenic/antiestrogenic activity and anti-inflammatory activity by producing alterations in cellular signaling [42]. They inhibit the expression of cytochrome p450 enzymes, which are involved in the process of activation of carcinogens. An increase in expression of phase II conjugating enzymes is facilitated by their excretion. Overexpression of phase II enzymes is associated with the toxicity of polyphenols [2].

Polyphenols affect pro-carcinogen metabolism by modifying the manifestation of cytochrome p450 enzymes involved in their stimulation to carcinogens. Carcinogen excretion may also be facilitated using these polyphenols by enhancing the phase II conjugating enzyme expression. Along with this, the stimulation of stage II enzymes can be caused by polyphenol toxicity [2]. The substrate of these enzymes is possible due to the formation of potentially toxic quinones. Improvisation of the body’s defense against xenobiotics is induced due to the self-detoxification of these enzymes by the intake of polyphenols [43]. Tea catechins proved its efficiency in cancer as it inhibits the alteration into cancer from high-grade prostate intraepithelial neoplasia lesions in men when given in the capsular form [44].

Along with this, polyphenols present in black tea (theaflavins and thearubigins) have good anticancer potential as they inhibit increase and proliferation in Du 145 prostate cancer cells [45]. The free radical scavenging potential of quercetin assists to show anticancer activity in lung cancer in mice induced by benzo(a)pyrene [46]. With this, inhibition of mutant p53 expression and apoptosis induction of treated cells is another potential of quercetin [47]. Resveratrol, a stilbene polyphenol, substantially proved for its anticancer potential via different in vitro and in vivo studies. Some of them are hepatocyte growth factor targeting and induction of apoptosis in human hepatocellular carcinoma (HCC), induction of cell death via the mediation of the epidermal growth factor receptor (EGFR) signaling pathway, regulation of AMP-activated protein kinase (AMPK) and increment in cell apoptosis induced by cisplatin [48–50]. Similarly, the antioxidant activity of resveratrol is helpful for its anticancer activity by modulation of various pathways like apoptosis, cell growth and inflammation [51]. Epigallocatechin gallate (EGCG), a major biologically active phenolic compound from green tea, also stated for its anticancer potential through different signal pathways [52]. Amyloid precursor protein (APP) acetylation and induction of apoptosis in human neuroblastoma, modulation of β-catenin activity and inhibition of head and neck cancer cell proliferation, rise in level of reactive oxygen species (ROS) with activation of caspase-3 and lowering the expression of vascular endothelial growth factor (VEGF) in esophageal squamous cells are some of the pathways for EECG’s anticancer action [53–55]. Curcumin, a major polyphenolic curcuminoid from turmeric rhizomes, exerts it anticancer potential via p53 pathway targeting in human osteosarcoma [56, 57]. Along with this, downregulation of Yes-associated protein expression in pancreatic cancer and extrinsic and intrinsic pathway triggering are other mechanisms for its anticancer action [58, 59]. In vivo studies of flavonoids like apigenin and chrysin and luteolin found in honey control the proliferation of pancreatic, glioma and aortic vascular smooth muscle cells in rats respectively [60–62].

#### Antiosteoporotic activity

Bone loss due to a deficiency of estrogen in menopausal women is considered to be a major cause of osteoporosis. Polyphenolic compounds like isoflavones show weak estrogenic action when observed in estrogen deficiency-induced rats or mice by ovariectomy. Loss of trabecular volume and bone density due to ovariectomy can be prevented by several weeks of dietary supplementation of daidzein, genistein and their glycosides [63–65]. Supplementation of soy proteins with reduced isoflavones shows antiosteoporotic activity in ovariectomized rats [66]. Inhibition of osteoclast cell differentiation, elevation in mineralization of bone, increase in alkaline phosphate action of osteoblast cells and decline in calcium stone formation induced by oxidative stress are the probable mechanisms for the antiosteoporotic activity of EGCG in rats [67–69]. Oleuropein, a polyphenol compound present in the olive leaf, acts via reduction of inflammatory biomarkers which may result in a decrease in bone loss in rats [70]. In vivo study of flavonoid fisetin showed the prevention of inflammation and bone loss in mice [71]. Resveratrol modulates SIRT1 (Sirtuin 1) activation and is responsible of its antiosteoporotic action [72]. Age-associated bone loss may be minimized by dietary intake of anthocyanin-rich berries due to their antioxidant potential via free radical scavenging [73]. In vivo antiosteoporotic study performed in female albino rats showed that ovarian hormone deficiency-induced bone loss has been prevented by aqueous black tea extract [74].

#### Cardioprotective effect

Numerous studies validated that the intake of polyphenols minimizes the risk of coronary heart diseases [75–77]. Atherosclerotic lesions developed in arteries remain clinically silent and then become active after decades and are responsible for the development of unstable angina, myocardial infarction or unexpected death [78]. Oxidation of low-density lipoprotein (LDL) is found to be the main mechanism in atherosclerosis development in the arteries, which is inhibited by the use of polyphenols [79]. Improvement in endothelial function, antiplatelet action, high-density lipoprotein (HDL), anti-inflammatory effects and antioxidant activity may be other mechanisms contributing to the protective effect of polyphenols in cardiovascular diseases. Quercetin found in an onion cause interruption in the formation of atherosclerotic plaques and inhibit the metalloproteinase I enzyme to reduce the mortality in patients with coronary heart diseases [42]. Various epidemiological investigations concluded that the risk of cardiovascular diseases like myocardial infarction is reduced due to the consumption of food enriched with polyphenols [80]. Polyphenols present in grape juice and red wine inhibit platelet aggregation, reduce bleeding time and exert antithrombotic effects [81]. Soy protein and green tea isolated from cocoa were found clinically effective for the decline in incidences of coronary heart disease and associated mortality via lowering of LDL and induction of nitric oxide-dependent vasodilation, respectively [82–84]. Clinical study of resveratrol revealed that 100 mg oral consumption for 12 weeks may support in the prevention of cardiovascular diseases and atherosclerosis via stimulation of endothelial function [85]. With this, it also modulates NO metabolism and helps for the improvement in vascular function in hypertensive and dyslipidemic patients [86].

#### Neuroprotective effects

Various neurodegenerative diseases, including Alzheimer’s disease, consist of damage to cellular components like DNA, lipids and proteins. In these conditions, oxidative stress is considered as a regulatory key factor. Intake of polyphenols may be responsible for the safeguarding of neurological diseases due to their strong antioxidant potential [87]. The onset of Alzheimer’s disease can be delayed by the intake of vegetables and fruit juices rich in polyphenols when taken three times per week [88]. The vital potential of vegetable and fruit polyphenols in neuroprotection plays an important role in influencing and modulating various cellular processes like proliferation, signaling, apoptosis and redox balance [89]. The risk of the development of Parkinson’s disease is reduced by the consumption of polyphenols in the form of green tea. These nutritional studies also revealed the protective role of polyphenols in Parkinson’s disease [90]. Tea consumption and incidence of neurodegenerative diseases show an inverse relationship due to its polyphenolic compounds including EGCG [91]. Curcumin found in turmeric showed its neuroprotective potential via reduction in Alzheimer’s disease pathogenesis [92]. Reduction in age-related cognitive impairment has been reduced by dietary intake of resveratrol in mice [93]. Additionally, resveratrol was also found efficacious in the prevention of blood-brain barrier impairment [94]. Quercetin has been reported for its protection in pheochromocytoma cell neurodegeneration induced by hydrogen peroxide [95]. In another study, mitochondria-targeted activities of quercetin were found to be a mechanism in protection against neurodegenerative diseases [96].

#### Antioxidant effects

Polyphenols are studied and recognized for their potential as natural antioxidant compounds for human health by combating and avoiding oxidative damage due to free radicals [97]. The hydroxycinnamic acid derivatives like caffeic acid and p-coumaric acid showed effective antioxidant activity against LDL peroxidation [98]. Ferulic acid which is a phenolic acid mostly found in oats, wheat and barley demonstrated prominent antioxidant activity and has been protective on human skin against UV rays [99, 100]. Quercetin, one of the important flavonoids, showed prominent antioxidant potential and is found as an effective, strong free radical scavenger in various in vitro research studies [101–103]. At an optimal dose of 1000 mg/day, rutin is a profound concentration-dependent free radical scavenger. Along with this, it is used in the management of hypertension, cancer and hypercholesterolemia [104–106]. Tea catechins including epigallocatechin gallate were found to be in vitro free radical scavengers. With this, these compounds were also found effective in decreasing protein carbonylation and lipid peroxidation in animal studies [107–109].

#### Others

Despite various health benefits of polyphenols mentioned above, they can be used in a few other health ailments too. Polyphenols are used in obstructive lung diseases like asthma [110, 111] as improvement in lung function in asthma patients was observed due to increased intake of genistein [112]. Polyphenols of tea minimize sunlight-induced skin damage, lipid peroxidation and erythema when given orally or applied topically studied in an animal study [113]. Theaflavins of black tea possesses antiviral action and shows anti-human immunodeficiency virus-1 (anti-HIV-1) activity. Theaflavin 3' gallate and theaflavin 3 3' digallate showed antiviral activity on the corona virus by inhibiting chymotrypsin-like protease [45].

### Classes of polyphenols

Polyphenolics is considered as one of the major classes of secondary metabolites consisting of more than 8000 polyphenolic compounds found in different plants. These phenolic compounds consist of shikimic acid as a close precursor and phenylalanine as a common intermediate. Conjugated forms of polyphenolic compounds were primarily found in which sugar residues are either linked with hydroxyl groups or directly to the aromatic carbon. These compounds also form conjugation with amines, organic acids, carboxylic acids, lipids and other phenolic compounds [114]. These compounds show profound protection against the development and worsening of several long-lasting pathological illnesses like aging, diabetes, cancer and cardiovascular problems (Table 1).

**Table 1:** Classes of polyphenols

Class	Main structure	Compound	Effects	Reference
Phenolic acids	Hydroxy-benzoic acid	p-Hydroxy-benzoic acid Gallic acid	Hypoglycemic Antimicrobial Antihypertensive Antihyperglycaemic	[115] [116] [117] [118]
	Cinnamic acid	Rosmarinic acid Ferulic acid Caffeic acid Chlorogenic acid	Hepatoprotective Nephroprotective Antihypertensive Antihyperglycaemic Neuroprotective Anti-inflammatory Antidiabetic Anti-inflammatory	[119] [120] [121] [122] [123] [124] [125] [126]
Flavonoids	Flavones	Chrysin Luteolin	Neuroprotective Cytotoxic Antiallergic Apoptotic	[127] [128] [129] [130]
	Flavanones	Naringenin Hesperetin Eriodictyol	Anti-inflammatory Antidiabetic Antiplatelet Apoptotic Hepatoprotective Anticancer	[131] [132] [133] [134] [135] [136]
	Flavonols	Quercetin Kaempferol Fisetin	Neuroprotective Antihypertensive Apoptotic Anti-inflammatory Cardioprotective Anti-inflammatory	[137] [138] [139] [140] [141] [142]
	Flavanols	Catechin Epicatechin	Neuroprotective Antioxidant Antidiabetic Nephroprotective	[91] [143] [144] [145]
Stilbenoids	–	Piceatannol Resveratrol	Antimutagenic Anticancer Apoptotic Immunomodulatory	[146] [147] [148] [149]
Lignans	–	Isotaxiresinol Secoisolariciresinol	Anti-osteoporotic Hepatoprotective Hepatoprotective Antioxidant	[150] [151] [151] [152]

**Fig. 1:**
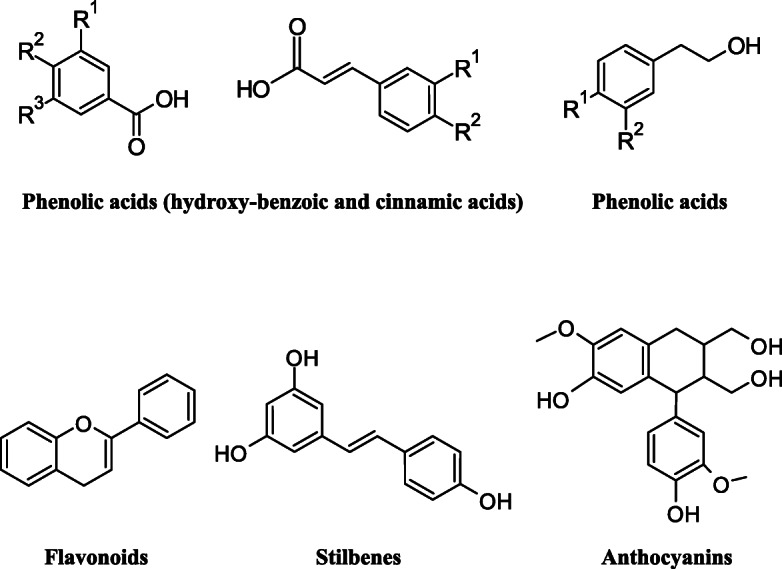
Chemical structure of the different classes of polyphenols

Polyphenolic compounds subdivided into subclasses like phenolic acids, phenolic alcohols, flavonoids, stilbenoids and lignans are given in Fig. 1 [3]. Out of these, most of the isolated, identified compounds are from the class of flavonoids.

#### Flavonoids

**Fig. 2:**
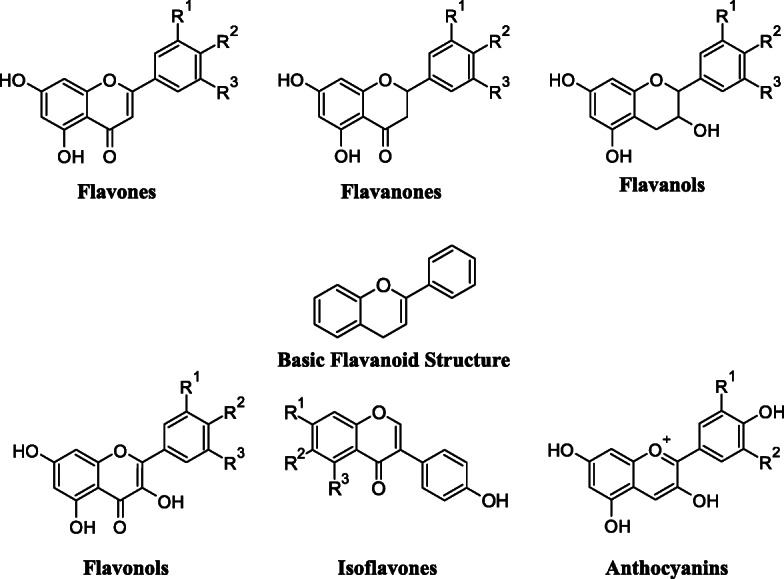
Chemical structure of classes of flavonoids

As earlier mentioned, flavonoids comprise the most isolated, identified and diversified class of polyphenolic compounds. Flavonoids are secondary plant metabolites that are responsible for the flower’s color and fragrance. Flavonoids are attributed to a wide range of health-promoting properties and are an integral part of many pharmaceutical nutraceuticals and medicinal and cosmetic formulations. They possess various pharmacological actions like antioxidant, antiviral, antibacterial, anti-inflammatory and anti-allergic potentials [153–155]. Flavonoids interact with several signal transduction pathways in the process of carcinogenesis, thereby reducing proliferation, angiogenesis and metastasis and increasing apoptosis [156]. To date, more than 6000 different flavonoids have been identified in plants and the list is continuously increasing [157]. They consist of a common diphenyl propane carbon skeleton along with two benzene rings A and B linked through the linear three-carbon chain (C6–C3–C6). Closed pyran ring C is formed by this central carbon chain. Variation of heterocycle involved, flavonoids may be classified into subclasses like flavones, flavanones, flavanols, flavonols (catechins and proanthocyanidins), anthocyanidins and isoflavonoids (Fig. 2 and Table 2). This classification depends on the existence or nonexistence of a double bond on the position 4 of the C ring and a double bond between C2 and C3 and the hydroxyl groups in the ring B.

**Table 2:** Classes of flavonoids

Flavonoid class	Examples	Source	Reference
Flavones	Chrysin	Honey, blue passion flower	[158]
	Luteolin	Common balm, parsley	[159, 160]
Flavanones	Hesperidin	Lemon, sweet orange	[161]
	Naringenin	Lemon, grapefruit	[162]
Flavonols	Quercetin	Apple, onion	[163]
	Kaempferol	Apple, onion	[163]
Flavanols	(+) Catechin	Green tea	[164]
	Epigallocatechin	Green tea	[164]
Anthocyanins	Cyanidin	Berries, grapes	[165]
	Delphinidin	Berries, grapes	[165]
Isoflavonoids	Daidzein	Soybeans	[166]
	Genistein	Soybeans	[166]

Flavone is a class of less common flavonoids consisting of a double bond between C2 and C3 in the heterocyclic ring of the flavan skeleton. A few of the important sources of flavones are celery and parsley. The skin of mandarin fruit also contains a large amount of polymethoxylated flavones.

##### Flavones

One of the flavonoids, flavones, are with a non-saturated 3-C chain and a double bond between C-2 and C-3, similar to flavonols, with which they vary in the lack of 3-position of the hydroxyl group. Flavones are commonly dispersed in the form of aglycones or glycosides among the higher plants. The distinction in composition between flavones and flavonols appears to have very significant implications in the roles of biogenesis, physiology and pharmacology and the phylogenetic and chemotaxonomic implications of these compounds [167]. Flavones are widespread as O-glycosides in biodiversity [168]. Several flavones such as chrysin, tangeretin and apigenin were researched for the therapy of experimental colitis; 30-day mice-supplemented apigenin feeding reduced dextran sulfate sodium (DSS)-induced colitis macroscopic and microscopic impairment [169]. In preclinical models, several flavones were studied for neuroprotection. In the streptozotocin-induced Alzheimer’s disease (AD) rat model, luteolin, a flavonoid discovered in celery, rosemary and parsley, has proved a definite neuroprotective impact that improves memory impairment and spatial learning. Apigenin, another prevalent flavone, has demonstrated comparable neuroinflammatory prevention activity. Apigenin-treated mice enhanced memory and learning capabilities by decreasing amyloid fibrillary deposits through modulation of beta-secretase 1. Luteolin in the liposomal form in olive fruit extract enhanced attention in kids with autism spectrum illnesses and brain fog in patients with mild cognitive consequences. Chrysin, a flavone found in multiple vegetables, fruits and mushrooms, has been suggested as a neurotrophic for nervous cells, anti-inflammatory and anti-amyloidogenic [170]. In addition, it also reduces the signs of DSS-induced colitis in mice by considerably diminishing colonic myeloperoxidase activity and decreasing proinflammatory cytokine, prostaglandin E_2_ (PGE2) and nitric oxide (NO) output [171].

##### Flavanones

Flavanones are usually glycosylated compounds with the disaccharide at C7 position and consisting of saturation between C2 and C3 and the presence of oxygen atom at the C4 position. Along with tomatoes and aromatic plants like mint, these compounds are abundantly present in citrus fruits. The aglycone flavanones naringenin, hesperetin and eriodictyol are present in grapefruit, oranges and lemons, respectively [172]. Compared to the glass of orange juice, whole citrus fruit contains five times more flavanone content as it is mostly accumulated in the spongy white portion and the segment separating the membranes of these fruits.

##### Flavonols

Flavonol is the most diverse class of flavonoids present in a food which is represented by a double bond between C2 and C2 with C3 position linked with a hydroxyl group. The representative compound from this class is quercetin. Flavonols are abundantly present in broccoli, onions, leeks and blueberries along with red wine and tea. It was observed that flavonol concentration differs among fruits or between different sides of the same fruit grown on the same tree. This type of effect was evident as the biosynthesis of flavonols is stimulated by sunlight. Probably due to sameness, flavonols are accumulated in the aerial and outer tissue of the fruits [173].

##### Flavanols

Flavanols are generally not available in the glycosylated form in foods consisting of the hydroxyl group at C3 position with saturation between C2 and C3. They occur in both the monomer (catechins) and the polymer (proanthocyanidins) forms. Epicatechin and catechin are the representative flavanols in fruit, while tea contains gallocatechin, epigallocatechin and epigallocatechingallate [174].

Cherry and apricots are the sources of catechins, while chocolate and green tea are the rich ones. Dimers, oligomers and polymers of catechins are also called condensed tannins or proanthocyanidins with varied structures and molecular weights. The range of degree of polymerization from 4 to 11 in cider apples is one representative example of the same [175]. The astringency of some fruits (berries, grapes) and beverages (beer, wine) and the bitter taste of chocolate is due to the proanthocyanidin content of the same [176].

##### Anthocyanins

Anthocyanins are abundantly and widely present in fruit skins, vegetables and cereals and are responsible for the different colors of fruits, flowers and vegetables [177]. The anthocyanin content of the fruit is generally proportional to the color intensity and ripening. Red wine is one of the sources in which these water-soluble pigments are present (up to 350 mg anthocyanins/L) which show the structural transformation on aging [178, 179]. Anthocyanins primarily occurs as glycosides which are called anthocyanidins formed from their respective aglycones. In this, the position of sugar moiety attachment is generally either at position 3 of C ring or 5, 7 position of A ring [180].

##### Isoflavones

Isoflavones are almost exclusively found in leguminous plants having a structural resemblance to estrogens. Glycitein, genistein and daidzein are isoflavone aglycones found in the soya plant which more often form conjugation with glucose [181, 182]. During storage and industrial processing, they often hydrolyze and form glycosides as they show sensitivity to heat [182].

## Conclusion

Polyphenols are naturally occurring secondary metabolites which is one of the most established categories of bioactive compounds. They constitute a broad repository of natural chemical diversity that includes a vast array of phytochemicals and enzymes. In humans, various scientific studies on consumable foods rich with these compounds revealed their potential health outcomes. They are found to be effective in the management of various chronic conditions like diabetes, cancer, cardiovascular diseases etc. Being a major subclass of polyphenol compounds and due to their widespread dietary distribution, flavonoids are believed to be non-toxic with little or no toxicity. This property is notable as numbers of phytochemicals in this class are available as a medicine in different dosage forms. So based on our literature review, we can conclude that the present understanding of phenolics and flavonoids will serve as the basis for the next scientific studies.

## Data Availability

Data and materials are available upon request.

## Abbreviations

AD: Alzheimer’s disease
AMPK: Adenosine monophosphate-activated protein kinase
ATP: Adenosine triphosphate
DNA: Deoxyribonucleic acid
DSS: Dextran sulphate sodium
EGFR: Epidermal growth factor receptor
HCC: Human hepatocellular carcinoma
HDL: High density lipoprotein
LDL: Low density lipoprotein
NO: Nitric oxide
PGE2: Prostaglandin E_2_
ROS: Reactive oxygen species
SIRT1: Sirtuin 1
VEGF: Vascular endothelial growth factor
